# Tart Cherry (*Prunus cerasus*) Extract Exerts High Intracellular ROS Scavenging Activity and Repression of ARE (Antioxidant Response Element) Pathway in Human Hepatocytes

**DOI:** 10.3390/ijms262210827

**Published:** 2025-11-07

**Authors:** Cécile Dufour, Mylène Rigal, Camille Gironde, Stephan Plattner, Christophe Furger

**Affiliations:** 1 Anti Oxidant Power (AOP), 78 Allées Jean Jaurès, 31000 Toulouse, France; cdufour@antioxidant-power.com (C.D.); mrigal@laas.fr (M.R.); cgironde@laas.fr (C.G.); 2 Iprona Lana SpA, Industriestraße 1/6, I-39011 Lana, BZ, Italy; stephan.plattner@iprona.com

**Keywords:** cellular assay, antioxidant activity, ROS, Nrf2-regulated ARE (Antioxidant Response Element) modulation, tart cherry, efficacy tests

## Abstract

Polyphenol-rich fruits represent promising natural candidates for mitigating oxidative stress. We determined in dose–response manner the intracellular antioxidant activities of *P. cerasus* (tart cherry) extract in HepG2 cells using three different cellular assays targeting specific mechanisms of action: (1) the AOP1 assay, to assess intracellular ROS scavenging activity; (2) the CAA assay, to estimate ROS scavenging activity at the cell membrane; and (3), the HepG2-ARE-luc assay, to evaluate Antioxidant Response Element (ARE) pathway modulation. Tart cherry extract exhibited a high and concentration-dependent intracellular ROS scavenging activity with the AOP1 assay (EC_50_ of 72.02 µg/mL), whereas antioxidant efficacy measured via the CAA assay was much lower (EC_50_ of 6.975 mg/mL). Notably, *P. cerasus* extract did not activate the ARE-driven luciferase gene expression. Instead, the extract induced a clear dose-dependent repression of ARE-driven transcriptional activity, with a reduction in luciferase gene expression ranging from 20 to 70% across the sample tested concentrations (0.38–98 µg/mL). These findings suggest that, at concentrations where it functions as a potent intracellular ROS scavenger, *P. cerasus* extract exerts a negative regulation of the ARE pathway. Further investigations are warranted to elucidate the compounds underlying these effects.

## 1. Introduction

Polyphenols are secondary metabolites produced by higher plants that exhibit a wide range of biologically valuable activities. Among these, their antioxidant capacity represents one of the most significant health-promoting properties. By modulating specific metabolic pathways and interacting with reactive oxygen and nitrogen species (ROS/RNS), as well as with the Nrf2-regulated Antioxidant Response Element (ARE) pathway, polyphenols influence the expression of key components in endogenous antioxidant systems. Through these mechanisms, they contribute to maintaining cellular redox balance and mitigating oxidative stress.

Oxidative stress arises from an imbalance between the generation of ROS and their neutralization, leading to the disruption of cellular homeostasis and potential damage to essential biomacromolecules such as lipids, nucleic acids, and proteins [1,2,3]. Both exogeneous factors (e.g., radiation, environmental pollutants, xenobiotics) and endogenous processes (e.g., cellular metabolism, aging, genetic factors) can elevate ROS production. Prolonged oxidative stress has been linked to aging and to the pathophysiology of chronic diseases including diabetes, cancer, neurodegenerative disorders, and cardiovascular complications [4,5]. Exercise represents another physiological condition in which oxidative stress plays a central role. During intense or prolonged physical activity, ROS production increases markedly, contributing to muscle fatigue, inflammation, and tissue damage [6,7].

Fruits and vegetables, rich sources of natural antioxidants, play a pivotal role in counteracting oxidative stress and reducing the risk of these chronic conditions [8,9,10,11]. Bioactive natural compounds with antioxidant properties have therefore attracted significant interest across the pharmaceutical, cosmetic and nutraceutical industries. Among these, drupe and berry fruits have gained particular attention due to their high levels of bioactive constituents such as polyphenols, fibers, minerals and vitamins. These fruits are especially rich in anthocyanins and have been extensively studied for their anti-inflammatory and antioxidant properties. Furthermore, epidemiological evidence suggests that regular consumption of berry polyphenols may confer protective health effects, including cardiovascular, neuroprotective, anticarcinogenic and antidiabetic benefits [12,13].

*Prunus cerasus* L., commonly referred to as tart cherry, belongs to the *Rosaceae* family and is recognized as a functional fruit of notable nutritional and phytochemical interest. Tart cherries constitute a rich source of polyphenolic compounds, with anthocyanins representing the predominant subclass of flavonoids. The principal anthocyanins identified in *P. cerasus* cultivars include cyanidin 3-glucosylrutinoside as the major component, followed by cyanidin 3-rutinoside, cyanidin sophoroside, and peonidin 3-glucoside. In addition to anthocyanins, *P. cerasus* contains other flavonoid classes, such as flavan-3-ols—particularly procyanidin B2 (a dimer of epicatechins)—and various flavonols, including rutin, isoquercitrin, kaempferol glycosides, and isorhamnetin glycosides. The predominant phenolic acids in tart cherry are neochlorogenic acid and chlorogenic acid. The in vitro antioxidant capacity of tart cherry extracts has been primarily attributed to cyanidin and its derivatives [14]. Tart cherries have been extensively investigated within the field of sports nutrition due to their potential recovery-enhancing properties. Numerous clinical trials have demonstrated that supplementation with tart cherry juice or concentrate can attenuate exercise-induced muscle soreness, inflammation, and oxidative stress following strenuous physical activity. For instance, marathon runners who consumed tart cherry juice prior to and after competition exhibited accelerated recovery and reduced concentrations of inflammatory cytokines compared with control groups [15]. Similarly, other studies have reported that tart cherry supplementation mitigates oxidative stress and inflammatory responses following repeated bouts of high-intensity cycling exercise [16] as well as reduces inflammatory and oxidative signaling in cell culture models [17]. Furthermore, a systematic review has highlighted the potential benefits of tart cherry supplementation for recovery, sleep quality, and exercise performance [18]. Collectively, these findings suggest that tart cherry polyphenols may play a beneficial role in modulating exercise-induced oxidative stress and promoting physiological recovery.

Studies investigating the in vitro antioxidant properties of tart cherry have traditionally relied on classical cell-free chemical assays, such as the Trolox Equivalent Antioxidant Capacity (TEAC), Oxygen Radical Absorbance Capacity (ORAC), and 2,2-diphenyl-1-picrylhydrazyl (DPPH) assays. However, these methodologies provide limited physiological relevance, as they do not account for factors such as bioavailability, cellular uptake, indirect antioxidant activity mediated through activation of endogenous defense pathways at gene expression level, or potential toxicity. In recognition of these limitations, the United States Department of Agriculture (USDA) discontinued the ORAC database in 2012, acknowledging that cell-free antioxidant assays do not accurately reflect antioxidant-related health benefits in humans [19].

To overcome the limitations of classical cell-free antioxidant assays, in vitro cell-based efficacy models have been developed to provide a more physiologically relevant assessment of biological antioxidant activity. In the present study, a standardized polyphenol-rich extract of *Prunus cerasus* was evaluated in a dose–response manner using human hepatocyte cells (HepG2). Three complementary cell-based assays were employed, each targeting a distinct cellular mechanism of antioxidant action: (1) the AOP1 assay, which measures intracellular reactive oxygen species (ROS) scavenging activity; (2) the CAA assay, which assesses ROS scavenging at the plasma membrane level; and (3) the ARE-driven luciferase reporter gene assay, which evaluates indirect antioxidant effects mediated through activation of cellular defense pathways at the transcriptional level. The AOP1 assay, in particular, relies on light-induced intracellular ROS generation to quantify antioxidant efficacy within living cells [20]. The AOP1 assay possesses the capability to discriminate between antioxidative, prooxidative, and cytotoxic effects, thereby providing a more comprehensive evaluation of cellular redox responses. Its principle is derived from the Light-Up Cell System (LUCS) approach, which was developed to assess cellular homeostasis [21]. Both the LUCS and AOP1 assays utilize a light-sensitive biosensor, thiazole orange (TO), which functions as a stressor and a fluorescent probe. Upon photoinduction, TO generates intracellular reactive oxygen species (ROS), allowing the quantification of antioxidant efficacy based on fluorescence modulation. The AOP1 assay has been successfully employed to classify the intracellular ROS scavenging capacities of purified antioxidant compounds as well as a variety of plant-derived extracts [20,22,23,24,25,26,27]. The Cellular Antioxidant Activity (CAA) assay utilizes the dichlorofluorescin diacetate (DCFH-DA) fluorescent probe in combination with a peroxyl radical generator (AAPH) as the oxidative stressor. This assay assesses the capacity of test compounds to scavenge lipid peroxyl radicals and thereby evaluate antioxidant activity at the plasma membrane level [28]. Finally, the HepG2-ARE-luciferase assay [29,30] was employed to evaluate the capacity of the *Prunus cerasus* extract to activate the antioxidant response element (ARE) pathway regulating the expression of ARE-driven genes encoding antioxidant and cytoprotective proteins. For comparative purposes, the chemical cell-free ORAC assay was also performed. This integrated approach aimed to provide a more comprehensive understanding of the antioxidant mechanisms of action exerted by *P. cerasus* polyphenols at the cellular level.

## 2. Results

### 2.1. Determination of Chemical Antioxidant Activity with a Cell-Free ORAC Assay

**Figure 1:**
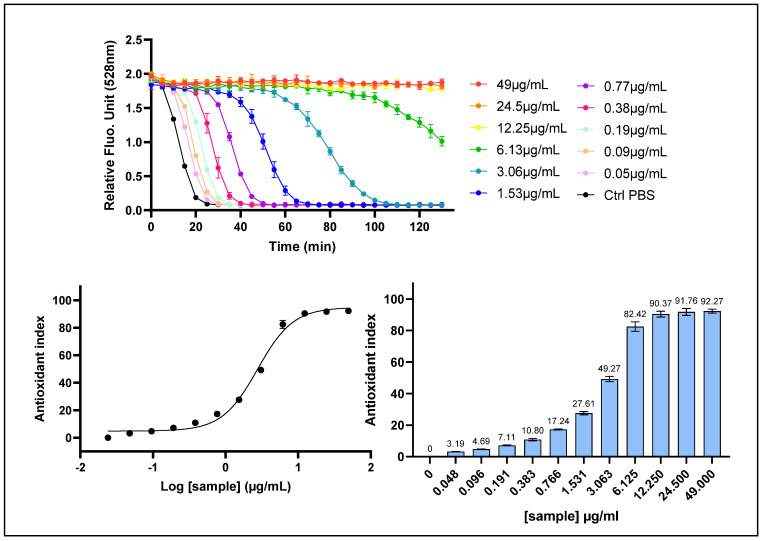
Chemical radical scavenging activity of *P. cerasus* by ORAC assay. *P. cerasus* (CherryCraft^®^) extract was assessed with ORAC assay on a dose–response manner. (**Upper panel**): kinetic profile of fluorescein fluorescence decay, where the x-axis represents time in minutes and the y-axis shows relative fluorescence unit (RFU) values recorded for eleven decreasing concentrations during 130 min. (**Lower panel**, **left**): dose–response curve, with the x-axis representing the log-transformed concentrations and the y-axis representing the ORAC Antioxidant Index values calculated with AUC method. (**Lower panel**, **right**): bar chart, with ORAC Antioxidant Index values given for each tested concentration. Data points: mean RFU values from triplicate measurements (n = 3); error bars: Standard Deviation (SD).

Prior to the antioxidant evaluation using the set of cell-based assays, the cell-free (or “chemical”) radical scavenging activity of the standardized *Prunus cerasus* fruit extract was determined using the Oxygen Radical Absorbance Capacity (ORAC) assay. The assessment was conducted in a dose–response manner, employing a series of eleven serial dilutions ranging from 49 to 0.05 μg/mL (Figure 1). In this assay, peroxyl radicals are generated through the thermal decomposition of 2,2′-azobis(2-amidinopropane) dihydrochloride (AAPH), which acts as a radical initiator to induce oxidation of the fluorescein probe. As the oxidation reaction progresses, fluorescein is progressively degraded, resulting in a decline in fluorescence intensity over time. The antioxidant capacity of the extract was quantified based on its ability to delay or inhibit the fluorescence decay, reflecting its radical scavenging potential.

The ORAC Antioxidant Index was calculated based on relative fluorescence unit (RFU) data using the area under the curve (AUC) method for each of the eleven tested concentrations. The results were presented using two complementary visual representations. In the lower left panel of Figure 1, normalized fluorescence data for each concentration were fitted to a sigmoidal dose–response model using GraphPad Prism 9 software, as described in Section 4, yielding an excellent fit (R^2^ = 0.9925). This dose–response relationship enabled the determination of the half-maximal effective concentration (EC_50_), corresponding to the concentration required to achieve 50% of maximal antioxidant activity, as well as EC_10_ and EC_90_ values. The best-fit EC_50_ value was 2.755 μg/mL, with a 95% confidence interval (CI) ranging from 2.447 to 3.096 μg/mL. The EC_10_ and EC_90_ values were estimated at 0.798 μg/mL (95% CI: 0.589–1.045) and 9.513 μg/mL (95% CI: 7.374–12.97), respectively. Alternatively, in the lower right panel of Figure 1, the ORAC Antioxidant Index was expressed for each tested concentration as the percentage of free radical scavenging activity.

The *Prunus cerasus* extract demonstrated potent ROS-scavenging capacity in the cell-free system, with EC_50_, EC_10_, and EC_90_ values determined within the microgram-per-milliliter range.

For comparison, Trolox, a water-soluble vitamin E analog commonly used as an antioxidant standard, was evaluated under identical ORAC assay conditions in a dose–response manner using nine concentrations ranging from 156.4 to 0.15 μg/mL. The resulting ORAC parameters for Trolox were as follows: EC_50_ = 13.86 μg/mL (95% CI: 11.86–16.33), EC_10_ = 3.396 μg/mL (95% CI: 2.275–4.793), and EC_90_ = 56.58 μg/mL (95% CI: 39.39–92.85), with an excellent model fit (R^2^ = 0.9888). When normalized to Trolox by calculating the EC_50_ (Trolox)/EC_50_ (*P. cerasus*) ratio, the *P. cerasus* extract exhibited approximately a five-fold greater antioxidant potency than Trolox, confirming its strong free radical scavenging efficiency in a chemical system.

### 2.2. Determination of Intracellular ROS Scavenging Activity with the AOP1 Cell-Based Assay

The intracellular ROS scavenging activity of *Prunus cerasus* was subsequently evaluated using the AOP1 assay in a dose–response manner with the human hepatocyte HepG2 cell line. In this cell-based model, oxidative stress is induced by repeated light applications that activate the biosensor, generating progressive ROS inside the cells and resulting in an increase in fluorescence intensity. The antioxidant efficacy of the tart cherry extract was quantified based on its ability to delay or suppress this fluorescence increase, reflecting a reduction in intracellular ROS accumulation.

The AOP1 Antioxidant Index was calculated based on relative fluorescence unit (RFU) data from the kinetic fluorescence profile for each of the decreasing concentrations using the area under the curve (AUC) method. The results were presented using two complementary visual representations: a dose–response curve in which the resulting data were fitted to a sigmoidal dose–response model using GraphPad Prism 9 software, as detailed in Section 4, and a bar chart in which the Antioxidant Index was expressed for each tested concentration as the percentage of intracellular ROS scavenging activity.

**Figure 2:**
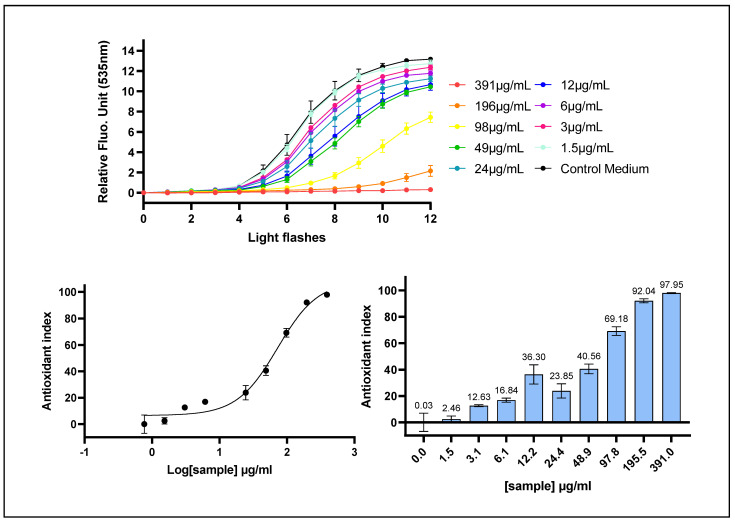
The intracellular ROS scavenging activity of *P. cerasus* was assessed on HepG2 cells using the AOP1 assay. HepG2 cells were incubated for 1 h with increasing concentrations of *P. cerasus* (CherryCraft^®^). (**Upper panel**): kinetic fluorescence profile, where the x-axis represents the light flash number and the y-axis displays the Relative Fluorescence Unit (RFU) values for each sample concentration. (**Lower panel, left**): Dose–response curve with the log-transformed concentration on the x-axis and the AOP1 Antioxidant Index calculated with the AUC method on the y-axis. (**Lower panel, right**): Antioxidant Index values for each tested concentration. Data points: mean RFU value from triplicate wells (n = 3); error bars: standard deviation (SD).

*Prunus cerasus* extract exhibited a clear concentration-dependent intracellular antioxidant activity, as evidenced by a progressive reduction in fluorescence intensity with increasing extract concentrations, culminating in near-complete suppression of the signal at the highest concentration tested (Figure 2, upper panel). The corresponding dose–response curve enabled the determination of an AOP1 EC_50_ value of 72.02 μg/mL (95% CI: 57.95–112.15), with EC_10_ and EC_90_ values of 15.80 μg/mL (95% CI: 8.631–23.56) and 328.3 μg/mL (95% CI: 183.4–1218), respectively, and an excellent model fit of R^2^ = 0.9787. Furthermore, no cytotoxic effects were observed following a 1 h exposure to any of the tested concentrations, including those as high as 25 mg/mL.

### 2.3. Determination ROS Scavenging Activity at Cell Membrane with the CAA (AAPH/DCFH-DA) Assay

The Cellular Antioxidant Activity (CAA) assay is based on the intracellular uptake of the fluorescent probe dichlorofluorescin diacetate (DCFH-DA), whose diacetate group enables passive diffusion across the plasma membrane. Once internalized, DCFH-DA is enzymatically deacetylated by intracellular esterases to form non-fluorescent dichlorofluorescin (DCFH). Upon exposure to oxidative stress, DCFH is oxidized to its fluorescent derivative, dichlorofluorescein (DCF), which serves as a quantitative indicator of intracellular ROS levels. In this assay, oxidative stress is chemically induced by 2,2′-azobis(2-amidinopropane) dihydrochloride (AAPH), a peroxyl radical generator that promotes peroxyl radicals and oxidation of DCFH at the plasma membrane interface, leading to increased fluorescence intensity. The antioxidant activity of the test sample is thus determined by its ability to attenuate this fluorescence increase through scavenging of peroxyl radicals.

**Figure 3:**
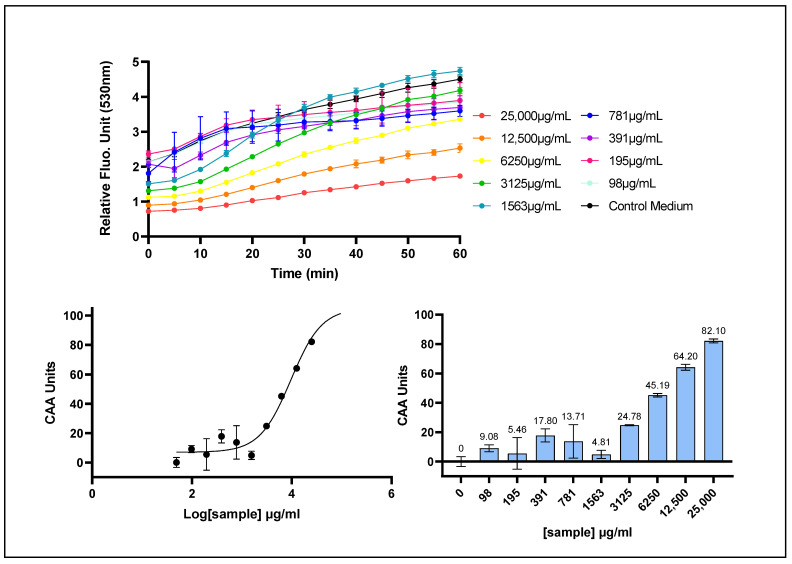
The cell membrane radical scavenging activity of *P. cerasus* was evaluated in HepG2 cells using the CAA (AAPH/DCFH-DA) assay. HepG2 cells were incubated for 1 h with decreasing concentrations of *P. cerasus* extract (CherryCraft^®^). (**Upper panel**): fluorescence emission kinetics of the DCFH probe; (**Lower panel**, **left**): dose–response curves, with the x-axis representing the log-transformed concentration and the y-axis the CAA Antioxidant Index values calculated with the AUC method. (**Lower panel**, **right**): CAA Antioxidant Index for each tested concentration. Data points: mean RFUs of triplicate wells; error bars: SD.

*Prunus cerasus* extract exhibited a clear concentration-dependent antioxidant response, as evidenced by its ability to scavenge reactive oxygen species (ROS) at the plasma membrane level in human HepG2 cells (Figure 3). The CAA assay yielded an EC_50_ value of 9.545 μg/mL (95% CI: 7.552–12.82), with corresponding EC_10_ and EC_90_ values of 1.982 μg/mL (95% CI: 1.254–2.857) and 45.97 μg/mL (95% CI: 27.12–101.2), respectively, with a fit giving an R^2^ value of 0.9697. The EC values obtained from the CAA assay were substantially higher than those derived from the AOP1 assay, indicating a comparatively lower antioxidant activity of the *P. cerasus* extract at the plasma membrane level.

### 2.4. Determination of ARE-Dependent Transcriptional Activity with a Hepatocyte-Based ARE Driven-Luciferase Reporter Assay

The capacity of *Prunus cerasus* extract to modulate gene expression through activation of the antioxidant response element (ARE) was evaluated using a stably transfected HepG2 cell line. This cell line harbors a luciferase reporter gene under the transcriptional control of the ARE sequence within its promoter region. Activation of the ARE sequence induces luciferase expression, resulting in the production of oxyluciferin (produced by luciferase protein activity) and a measurable luminescent signal. This assay enabled quantitative assessment of the ability of tart cherry extract to induce ARE-driven genes involved in cellular antioxidant defense mechanisms.

**Figure 4:**
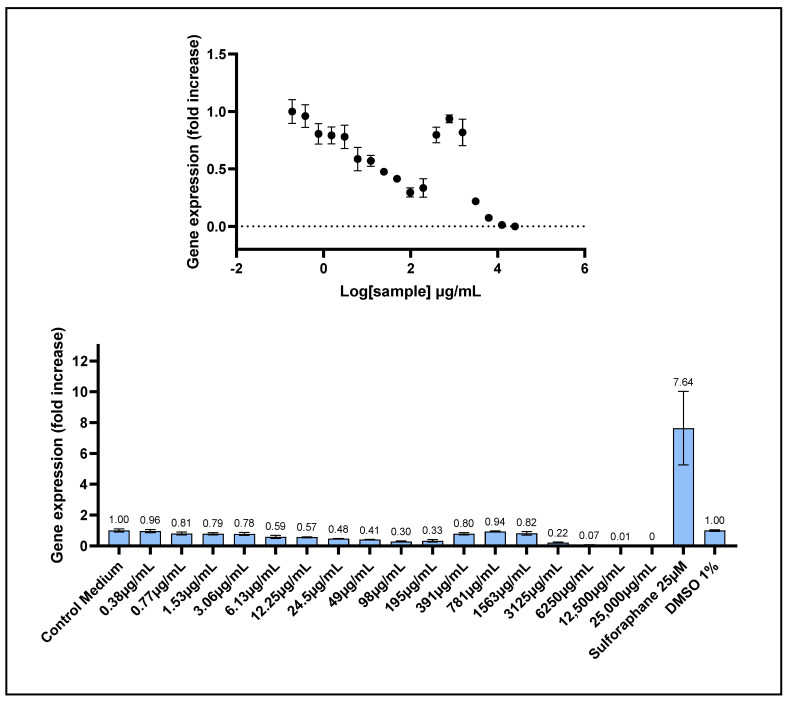
ARE transcriptional activity of *P. cerasus* was assessed using a luciferase reporter gene assay in ARE-luc-HepG2 cells. ARE-luciferase-HepG2 cells were treated for 17 h with a range of decreasing concentrations (from 25,000 μg/mL to 0.38 μg/mL) of *P. cerasus* (CherryCraft^®^) and oxyluciferin luminescence was measured as relative luminescence units for each sample concentration. (**Upper panel**): dose–response curve, in which the log-transformed concentrations on x-axis are plotted against gene expression fold increase. (**Lower panel**): luciferase gene expression represented as gene expression fold increase (FI) for each tested concentration, relative to vehicle controls and positive control (sulforaphane, 25 μM; FI = 7.64). Error bars: SD values based on n = 6 for sulforaphane and DMSO, n = 4 for control medium, 49 and 98 µg/mL and on n = 2 otherwise.

The *Prunus cerasus* extract did not induce activation of the antioxidant response element (ARE) pathway in the reporter gene assay (Figure 4). Instead, it exhibited a clear dose-dependent repression of ARE activity. This down-regulatory effect was observed at concentrations ranging from 0.77 to 195 μg/mL, reaching a maximum inhibition of approximately 70% at 98 μg/mL. Interestingly, ARE-driven gene expression partially recovered toward control levels at intermediate concentrations between 391 and 1563 μg/mL, before declining to zero at higher concentrations, an effect typically associated with cytotoxicity. The dose–response curve did not allow us to determine an EC_50_ value. However, these findings demonstrated a dose-dependent biphasic modulation of the ARE pathway by the tart cherry extract.

## 3. Discussion

In this study, the intracellular antioxidant activity of the anthocyanin-rich *Prunus cerasus* extract was evaluated using a panel of three cell-based bioassays, each targeting distinct mechanisms of action: direct ROS scavenging either intracellularly (AOP1 assay) or at the plasma membrane (CAA assay), and indirect antioxidant activity via modulation of ARE-driven gene expression. The half-maximal effective concentration (EC_50_) of the extract varied considerably across the different assays and mechanisms. Specifically, EC_50_ values were markedly higher in the CAA assay (9.975 mg/mL), indicating comparatively low antioxidant activity at the plasma membrane, whereas much lower EC_50_ values were observed in the AOP1 assay (72 μg/mL), reflecting higher intracellular ROS scavenging efficacy. In comparison, the classical cell-free ORAC assay yielded EC_50_ values in the low microgram per milliliter range (2.755 μg/mL). These findings underscore the necessity of employing reliable, sensitive, and mechanistically well-characterized cell-based antioxidant assays to accurately assess the biological activity of compounds in physiologically relevant cellular contexts.

The efficacy concentrations determined using the AOP1 assay (EC_10_ ≈ 15.8 μg/mL) and the lower end of the concentration range in the ARE-luciferase assay (0.77 μg/mL, corresponding to a 20% reduction in luciferase expression) may approximate physiologically relevant intracellular levels. Such concentrations could potentially be achieved in vivo following supplementation with tart cherry concentrate. Supporting this, a human study administering a pure synthetic ^13^C-labeled anthocyanin (cyanidin 3-glucoside, 500 mg) to volunteers detected 25 distinct ^13^C-labeled phenolic metabolites in serum, reaching a total peak concentration of 5.97 ± 2.14 μM [31]. Similarly, in a randomized controlled trial involving 12 healthy male participants, consumption of tart cherry concentrate (30 mL or 60 mL) resulted in plasma phenolic acid concentrations in the range of ~0.2–0.5 μg/mL, with chlorogenic acid reaching ~0.2–0.4 μg/mL and vanillic acid ~0.3–0.5 μg/mL [32]. Notably, protocatechuic acid, a major anthocyanin metabolite, reached a peak plasma concentration of approximately 2.75 μg/mL, with no significant difference observed between doses, whereas vanillic acid peaked at ~0.30 μg/mL. These bioavailability data may suggest that, although the EC values determined in our study reflect the activity of the whole *Prunus cerasus* extract rather than individual compounds, the concentrations required for antioxidant activity could be broadly consistent with those achievable in vivo. For instance, considering the extract’s composition (19.69% total polyphenols and 3.29% anthocyanins; Supplementary Table S1), the AOP1 EC_10_ value of 15.80 μg/mL corresponds to approximately 2.95 μg/mL of total polyphenols and 0.52 μg/mL of anthocyanins. This may align with the ~2.75 μg/mL C max of protocatechuic acid observed in humans [32], supporting the physiological relevance of the intracellular antioxidant activity measured in the AOP1 assay.

Beyond its potent intracellular antioxidant activity, *Prunus cerasus* extract exhibited a dose-dependent repression of ARE-driven transcriptional activity, as evidenced by a reduction in luciferase expression ranging from 20% to 70% across the 0.77–98 μg/mL concentration range. The concentrations at which ARE downregulation was observed corresponded closely with those eliciting significant intracellular ROS scavenging in the AOP1 assay (EC_10_ and EC_50_ values of 15.80 and 72.02 μg/mL, respectively). A plausible explanation for this effect is that the pronounced radical scavenging activity of the extract substantially reduced intracellular ROS levels, potentially triggering a cellular feedback mechanism that downregulates ARE pathway signaling. The specific phenolic constituents responsible for this ARE repression remain to be identified. Notably, previous studies with polyphenolic plant extracts have reported either activation or no effect on the ARE pathway using this HepG2-ARE-luciferase assay [23,25,29,30]. To our knowledge, this study is the first to demonstrate a clear dose-dependent repression of the ARE pathway by tart cherry extract.

The activation of the antioxidant response element (ARE) pathway by pro-oxidant stimuli is a well-established cellular defense mechanism against oxidative stress, with nuclear factor erythroid 2–related factor 2 (Nrf2) serving as the key transcription factor. Nrf2 activity is tightly regulated at multiple levels, including epigenetic, transcriptional, translational, and post-translational mechanisms [33,34]. Nrf2 mediates the activation of gene expression by forming heterodimers with MAF proteins in the nucleus, which then bind to ARE sequences located in the regulatory regions of hundreds of target genes. These genes encode proteins involved in a wide range of cellular processes, including redox homeostasis, drug and xenobiotic metabolism, heme metabolism, NADPH generation, the pentose phosphate pathway, autophagy, fatty acid oxidation, lipase activity, transcriptional regulation and Keap1 expression [35,36]. A key regulatory mechanism of Nrf2 transcriptional activity is its stabilization, which is controlled by Kelch-like ECH-associated protein 1 (Keap1). Keap1 functions as a sensor protein, containing highly reactive cysteine residues that undergo oxidative modifications in response to elevated intracellular ROS levels [37]. Under basal conditions, when cellular oxidative stress is low or moderate, Nrf2 is maintained in the cytosol at relatively low levels due to Keap1-mediated targeting for ubiquitination and subsequent proteasomal degradation. Upon oxidative stress, structural modifications of the Keap1–Nrf2 complex allow Nrf2 to translocate to the nucleus, where it binds to ARE sequences and activates transcription of genes encoding antioxidant and cytoprotective proteins. Several phytochemicals capable of activating the ARE pathway have been identified, including polyphenols such as curcumin, resveratrol, quercetin, genistein, and andrographolide [38]. Most of these phytochemicals act as electrophilic oxidants [39]; however, an alternative mechanism of Keap1–Nrf2 dissociation has been described, in which Keap1 interacts with non-electrophilic compounds to release Nrf2 and allow its nuclear translocation [40]. Upon resolution of oxidative stress, Keap1 contributes to the deactivation of the ARE pathway. Through its nuclear export sequence (NES), Keap1 mediates the export of Nrf2 from the nucleus to the cytosol, where Nrf2 is subsequently targeted for ubiquitin-mediated proteasomal degradation [41,42].

Beyond the Keap1–Nrf2 interaction, multiple regulatory mechanisms modulate ARE pathway activation and deactivation at various levels. These include interactions with other transcription factors, such as Bach1, which antagonizes Nrf2 binding to ARE sequences, as well as kinase-mediated modifications that can enhance or reduce Nrf2 stability and nuclear translocation. Additional post-transcriptional regulation by microRNAs can also suppress Nrf2 expression. In our HepG2-ARE-luciferase screening assay, only the ability of the *Prunus cerasus* extract to modulate ARE-driven gene expression was addressed, and a repression effect was not anticipated. The molecular mechanisms underlying this observation remain to be elucidated and may involve processes such as altered Nrf2 nuclear translocation or Bach1-mediated antagonism. Nevertheless, negative transcriptional regulation of the ARE pathway may represent a fundamental mechanism for controlling gene expression. Notably, Liu et al. (2018) reported that certain genes containing ARE sequences are subject to direct repression in an ARE-dependent manner [43]. These authors identified an Nrf2–RPA1 complex that binds to a specific seven-nucleotide sequence (termed NRE), located adjacent to ARE sequences in the promoter regions of certain genes. The NRE facilitates recruitment of replication protein A1 (RPA1), which prevents Nrf2 from forming heterodimers with MAF proteins—a necessary step for ARE binding and transcriptional activation. Using this mechanism, a total of 55 genes were identified as potentially negatively regulated by the Nrf2–RPA1 complex. Among these, MYLK, encoding the non-muscle isoform of myosin light chain kinase, plays a critical role in cytoskeletal actin regulation and vascular integrity. Notably, repression of MYLK attenuated inflammatory lung injury in mice, suggesting that negative regulation via the Nrf2–RPA1 complex may contribute to the mitigation of adverse inflammatory responses. Additional genes negatively regulated by Nrf2 include those encoding the pro-inflammatory cytokines IL-1β and IL-6, as well as NADPH oxidase 4 (NOX4) [34].

Inhibition of the ARE pathway may be particularly relevant in the context of cancer. Many tumor types exhibit Nrf2 overexpression and constitutive activation of the ARE pathway, which can confer a survival advantage and contribute to resistance against chemotherapy and radiotherapy. Accordingly, the identification of natural compounds capable of inhibiting ARE signaling may provide a strategy to sensitize cancer cells to anticancer therapies. For example, brusatol, a triterpene lactone isolated from *Brucea javanica*, has been shown to repress the ARE pathway and enhance the efficacy of therapeutic drugs [44]; however, its mechanism of action is not specific, as it broadly inhibits protein translation [45]. Within the flavonoid family, several compounds have been reported to modulate the ARE pathway, with potential implications for overcoming chemoresistance in cancer cells. Luteolin, for instance, suppressed ARE activity and counteracted chemoresistance in lung cancer cells [46]. In a screening study of 12 common flavonoids, only luteolin exhibited a dose-dependent inhibitory effect on ARE-luciferase activity, reducing luciferase expression to 0.8-, 0.65-, and 0.4-fold of control at 1, 3, and 10 μM, respectively, [46]: Epigallocatechin gallate (EGCG) has also been reported to inhibit ARE signaling at 200 μM [47]. Wogonin, a flavonoid isolated from the root of *Scutellaria baicalensis*, inhibited the ARE pathway by increasing Nrf2 transcript instability and was able to reverse drug resistance in breast cancer cells [48]. More recently, corilagin, an ellagitannin, was shown to induce apoptosis and downregulate the ARE pathway in glioma cells overexpressing Nrf2 [49]. However, the role of these compounds remains controversial, as subsequent studies have suggested that luteolin, EGCG, and wogonin may also act as ARE activators, highlighting the complexity of their effects on ARE pathway modulation [50,51,52].

Redox processes form the foundation of exercise physiology, as they underlie the signaling pathways that regulate both acute exercise-induced responses and chronic adaptive processes [53]. Both acute and chronic exercise have been shown to activate Nrf2-regulated ARE signaling, which contributes to the upregulation of endogenous antioxidant defenses. However, the influence of antioxidant supplementation on these pathways remains debated. Some studies have reported that antioxidant treatments may attenuate exercise-induced activation of antioxidant enzymes and related proteins (“blunt” effect), whereas others have found no significant effects of antioxidant supplementation in humans. Notably, tart cherry juice consumption has been associated with improved recovery following strenuous exercise. In one study, participants who consumed tart cherry juice for five days prior to and two days following a marathon exhibited accelerated post-exercise recovery, characterized by reduced lipid peroxidation and attenuated inflammatory responses, including a smaller increase in circulating IL-6 levels, compared to the placebo group [15]. Interestingly, the cytokines IL-6 and IL-1β are encoded by genes that are negatively regulated by Nrf2 [34]. The ARE repression effect observed in our study—with *Prunus cerasus* extract inducing up to a 70% reduction in gene expression at 98 μg/mL—may suggest that downregulation of this pathway could contribute to improved post-exercise recovery. This may align with previous observations showing reduced circulating IL-6 levels in athletes consuming tart cherry juice. However, it is important to note that Nrf2-mediated inhibition of cytokines such as IL-6 and IL-1β can occur through mechanisms that are either dependent on or independent of the ARE binding motif, depending on the cellular context [54]. Taken together with human bioavailability kinetics (peak plasma concentrations observed approximately 1–3 h post-ingestion, with detectable signals up to ~8 h), these findings may help define practical pre- (~1–2 h) and post-exercise intake windows to effectively target the early oxidative and inflammatory phases of recovery.

In the present study, *Prunus cerasus* extract exhibited antioxidant activities through distinct mechanisms of action, as demonstrated by a set of live-cell assays showing both strong intracellular ROS scavenging activity and a dose-dependent repression of the ARE pathway. Collectively, these cellular findings—when considered alongside existing evidence from human bioavailability and exercise intervention studies—support the hypothesis that tart cherry extracts may aid post-exercise recovery by mitigating oxidative stress and potentially attenuating pro-inflammatory signaling.

## 4. Materials and Methods

### 4.1. Plant Material

The tart cherry (*Prunus cerasus* L.) extract is a proprietary ultra-filtered natural extract with the brand name CherryCraft^®^ obtained from the Nadwiślanka variety, also known as Vistula Cherry, and is produced by Iprona Lana SpA, Italy (product number: B0330082 CherryCraft^®^ 14% L2201304749). See the component identification data of CherryCraft^®^ in Table 1 and in Table S1 (Supplementary Data) for phenolic composition of the extract. HPLC analysis was performed on a reverse-phase C18 column with UV–visible detection at 520 nm. Full chromatographic conditions are available upon request from the manufacturer (Iprona, Lana, Italy). Stability of the extract is documented by similar HPLC chromatograms obtained from different batches (Figure S1, Supplementary Data).

### 4.2. Chemicals and Cell Lines

Trolox, Fluoroscein, Sulforaphane (SFN), thiazole orange (TO), 2,2-azobis(2-methylpropionamidine) dihydrochloride (AAPH) and 2′,7′-dichlorofluorescin diacetate (DCFH-DA) were obtained from Sigma-Aldrich (Saint-Quentin Fallavier, France). Gibco DMEM (high glucose, Gluta-MAX supplement and pyruvate), Fetal Bovine Serum (FBS) (HyClone Laboratories, Logan, UT, USA), penicillin–streptomycin solution (100X) (Gibco), 0.05% Trypsin-EDTA (HyClone), Gibco Selective Antibiotic Geneticin (G418) (50 mg/mL) and Gibco DPBS without Calcium and Magnesium (1X) were procured from Thermo Fisher Scientific (Illkirch-Graffenstaden, France). The HepG2 cell line (catalog number HB8065) was obtained from the American Type Cell Collection (ATCC) (LGC Standards, Molsheim, France). ARE Reporter–HepG2 cell line (catalog number 60513) was acquired from BPS Bioscience (San Diego, CA, USA).

### 4.3. Cell Culture Conditions

Human hepatocytes from the HepG2 cell line (passages 17 to 25) were cultured at 37 °C with 5% CO_2_ in GlutaMAX supplemented DMEM, complemented with 10% FBS and 1% penicillin–streptomycin (1X) solution. ARE Reporter–HepG2 cells (passages 5 to 17) were cultured under the same conditions with the addition of 0.6 mg/mL of Geneticin to the medium. Once the cells reached 70–80% confluence, they were transferred into clear-bottom 96-well microplates for 24 h. Cells were plated at 75,000 cells/well (75 μL/well).

### 4.4. Preparation of Samples

*P. cerasus* extract were kept at 4 °C and solubilized at final concentration of 50 mg/mL in DMEM culture medium without FBS. Sample was centrifuged at 8700 rpm 10 min. No pellet was present and the solubilization was complete. For dose–response studies, decreasing concentration ranges were made from the 50 mg/mL solution using serial dilutions in serum-free DMEM.

### 4.5. Chemical Radical Scavenging Activity with ORAC Assay

Serial dilutions of *P. cerasus* extract (11 different concentrations) or of Trolox were mixed with fluorescein solution (50 nM). AAPH solution (5 mM) was added to initiate the radical production. The decreasing fluorescence intensity of fluorescein [485 nm (ex)/528 nm (em)] was assessed using a Varioskan Flash Spectral Scanning Multimode Reader (Thermo Fisher Scientific, Waltham, MA, USA). Relative fluorescence Unit (RFU) was measured every five minutes for 2 h at 37 °C. The ORAC antioxidant index was calculated from the kinetic area under curve (AUC) and normalized using the formula: ORAC index = (AUC_sample_ − AUC_control_)/(RFU_t0 of control_ × total time in min − AUC_control_) × 100, in which controls were PBS for plant extract and 1% EtOH for Trolox. Two independent experiments were performed with nine decreasing concentrations of extract and triplicate wells per concentration.

### 4.6. Intracellular ROS Scavenging Activity with AOP1 Assay

The AOP1 bioassay (patented technology) measures the ability of compounds to scavenge intracellularly generated reactive species using a photosensitive biosensor [21]. The antioxidant effect is assessed by monitoring the delay in the kinetic progression of biosensor fluorescence emission [20]. Cells were incubated in fresh serum-free DMEM with a range of extract decreasing concentrations for 1 h at 37 °C in a 5% CO_2_ environment. Two independent experiments were performed (with nine decreasing concentrations for each extract) and triplicate wells per concentration. Following the 1 h incubation with *P. cerasus* extract, the cells were treated with the biosensor for an additional 1 h at 37 °C in 5% CO_2_ using serum-free medium to avoid interactions with serum components. The Relative Fluorescence Unit (RFU) was measured at 535 nm using a Varioskan Flash Spectral Scanning Multimode Reader (Thermo Fisher Scientific, Waltham, MA, USA) following repeated 470 nm LED illuminations applied across the entire 96-well plate. Raw kinetic profiles were recorded during each illumination and fluorescence reading sequence (20 iterations). The cellular Antioxidant Index (AI) was calculated from normalized kinetic profiles as follows: AI (%) = 100 − 100 (_0_∫^20^ RFU_sample_/_0_∫^20^ RFU_control_), where the control was the cell culture medium only or the medium with solvent only. The Antioxidant Index (AI) was plotted against the logarithm of the sample concentration (Log) and fitted to a sigmoid model based on the equation AI = AI_min_ + (AI_max_ − AI_min_)/(1 + 10(Log(EC_50_/SC) × HS)), where SC represents the sample concentration, HS is the Hill slope (or tangent slope at the inflexion point), and EC_50_ is the concentration that achieves 50% of the maximal effect (or the half maximal effective concentration). Dose–response curves and the corresponding EC_50_, EC_10_ and EC_90_ were calculated using Prism 9 software (GraphPad, San Diego, CA, USA). The best-fit EC_50_ values were determined with 95% confidence intervals using the asymmetrical likelihood method. Coefficients of determination (R^2^) were greater than 0.97 for the calculation of EC values. Two independent experiments were conducted in serum-free culture medium (with nine decreasing concentrations of extract) and in triplicate wells for each concentration.

### 4.7. ROS Scavenging Activity at Cell Membrane with CAA Assay (AAPH/DCFH-DA)

The CAA assay [28] is based on the cell uptake of the DCFH-DA probe (2′,7′-dichlorofluoresceindiacetate) through the plasma membrane, being facilitated by its diacetate group. Once inside the cell, the DCFH-DA is deacetylated to non-fluorescent DCFH, which becomes fluorescent upon its oxidation to DCF. In this assay, oxidation was initiated by the radical generator AAPH (2,2′-azobis (2-amidinopropane) dihydrochloride, which induced the production of peroxyl radicals at the plasma membrane, converting DCFH to its fluorescent product, DCF. The cells were incubated with nine decreasing concentrations of the extract (25 mg/mL to 98 μg/mL) for 1 h at 37 °C in 5% CO_2_ in the presence of DCFH-DA (30 μM). Following incubation, the cells were washed three times, and AAPH was added (600 μM). Fluorescence was measured using a Varioskan Flash Spectral Scanning Multimode Reader (Thermo Fisher Scientific, Waltham, MA, USA). Relative fluorescence unit (RFU) was measured every 5 min, and the readings continued for 2 h. Dose–response curves were calculated using the formula: CAA Units = 100 − (_0_∫^50^ RFU_sample_/_0_∫^50^ RFU_control_) × 100. Two independent experiments were conducted in serum-free culture medium (with nine decreasing concentrations of extract) and in triplicate wells for each concentration.

### 4.8. Transcriptional Activity of the Nrf2-Regulated ARE Pathway with HepG2-ARE-Luc Assay

The ability of the samples to activate the Nrf2-regulated ARE pathway at different concentrations was assessed using a luciferase reporter gene assay, as previously described [25,29,30]. Stably transfected ARE-luc-HepG2 cells were incubated for 17 h at 37 °C in 5% CO_2_ with nine decreasing concentrations of *P. cerasus* extract. Following incubation, the cells were treated with a mixture of cell lysis buffer and luciferin (the substrate of luciferase) (BPS Bioscience, San Diego, CA, USA). Luminescence was measured using a Varioskan Flash Spectral Scanning Multimode Reader (Thermo Fisher Scientific, Waltham, MA, USA) to determine the Relative Luminescence Units (RLUs), reflecting luciferase gene expression via the activation of the ARE (Antioxidant Response Element) sequence. Sulforaphane (SFN) was used as a positive control. The results were expressed as the fold increase (FI) relative to the negative control at t = 20 min using the formula FI = (RLU_sample_/RLU_control_). FI values were plotted against the logarithm of the sample concentration. Two independent experiments were performed with each concentration in duplicates, to cover the full range of concentrations.

## 5. Conclusions

The antioxidant activities of the anthocyanin-rich *Prunus cerasus* (tart cherry) extract were evaluated in HepG2 cells using a set of complementary cellular assays addressing specific antioxidant mechanisms: direct scavenging of intracellular ROS using the AOP1 assay, scavenging of lipid peroxides at the plasma membrane via the CAA assay, and indirect modulation of antioxidant defense through ARE-driven gene expression. A summary of the antioxidant activities measured across these assays, together with a complementary cell-free ORAC test, is presented in Table 2.

*P. cerasus* extract exhibited a marked intracellular ROS scavenging activity in HepG2 cells, as determined by the AOP1 assay (EC_50_ = 72 µg/mL). Interestingly, at concentrations where potent intracellular ROS scavenging was observed, the extract also acted as a repressor of the ARE pathway. This repression may result from a pronounced decrease in intracellular ROS levels or from alternative molecular interactions affecting the cellular antioxidant response machinery.

These findings emphasize the multifaceted biological roles of polyphenols in modulating redox homeostasis. The observed dual activity—strong direct ROS scavenging combined with repression of ARE signaling—illustrates the complexity of antioxidant regulation and underscores the importance of assessing both direct and indirect mechanisms when characterizing the cellular effects of polyphenol-rich extracts.

## Figures and Tables

**Table 1:** Component identification of *P. cerasus* extract.

Analysis	Method	Unit	Value
pH	Powder—diluted 1:5 in deionized water		3.35
Total acidity (W) pH 7.0	IFU 3	g/kg	38.86
Total acidity (Z) pH 8.1	anhydrous, IFU 3	g/kg	42.859
Dry matter	Drying at 105 °C	%	99.44
Moisture	Drying at 105 °C	%	0.56
Density (measurement)	Bulk density	g/mL	0.529
Color	520 nm, pH 1.0		2581.02
Color	420 nm, pH 1.0		896.86
Color	Ratio 520 nm/420 nm		2.878
Anthocyanins	as Cya-3-glu (spectrometric by pH-difference)	g/kg	32.943
Anthocyanins	as Cya-3-glu (HPLC)	g/kg	29.5
Polyphenols	as Catechin (Folin–Ciocalteu)	g/kg	196.907
Yeasts	Pour Plate	cfu/g	<5
Molds	Pour Plate	cfu/g	5
TVC (total viable count)		cfu/g	55
Coliforms	Product	cfu/g	0
*E. coli*		cfu/g	0
Enterobacteriaceae		cfu/g	0
Sucrose	IFU 56	g/kg	0
Glucose	IFU 55	g/kg	88.75
Fructose	IFU 55	g/kg	75.88
Total sugars		g/kg	164.63
L-Malic acid	IFU 21	g/kg	24.84
Citric acid	IFU 22	g/kg	0.05
D-Isocitric acid	IFU 54	mg/kg	40.39
Galacturonic acid	HPLC	mg/kg	269.00
Sodium (Na)	ICP-MS	mg/kg	67.7
Potassium (K)	ICP-MS	mg/kg	3064.21
Calcium (Ca)	ICP-MS	mg/kg	454.44
Phosphorus	as phosphate (ICP-MS)	mg/kg	1641.628
Magnesium (Mg)	ICP-MS	mg/kg	227.99
L-Malic acid (amount)		g	0.639
D-Isocitric acid (amount)		mg	1.039
Ash		g	0.226
Potassium (amount)		mg	78.853
Calcium (amount)		mg	11.694
Magnesium (amount)		mg	5.867
Phosphate (amount)		mg	42.245
Sulfate (amount)		mg	6.176
Sum of free amino acids	HPLC	mg/kg	4373
Hydroxycinnamic acids	Chlorogenic acid (HPLC)	mg/kg	1297
Hydroxycinnamic acids	Neochlorogenic acid (HPLC)	mg/kg	927
Flavonols	Rutin (Quercetin-3-Rut) (HPLC)	mg/kg	2710
Flavonols	Isoquercitrin (Quercetin-3-Glu) (HPLC)	mg/kg	179
Flavonols	Kaempferol-3-Rut (HPLC)	mg/kg	442
Flavonols	Isorhamnetin-3-Rut (HPLC)	mg/kg	1043
Flavonols	Quercetin (HPLC)	mg/kg	32.4
Flavonols	Ratio Rutin/Isoquercitrin		15.14
Proanthocyanidin	Procyanidin B2 (HPLC)	mg/kg	3965
Energy (kcal)	kcal	kcal/100 g	372
Energy (kJ)	kJ	kJ/100 g	1577
Fat	total fat	g/100 g	0
Carbohydrates	total carbohydrates	g/100 g	88.87
Protein	N × 6.25	g/100 g	1.4
Dietary fiber	total	g/100 g	5.36

**Table 2:** Antioxidant activities of *P. cerasus* extract. EC_10_, EC_50_ and EC_90_: efficacy concentrations required to achieve 10%, 50% and 90% of the maximal antioxidant activity; [95% CI]: 95% confidence interval; R^2^: coefficient of determination for dose–response sigmoid fits; ND: EC values could not be determined (no sigmoid fits).

*Assay (Mechanism)*	*EC*_50_ (µg/mL) [95% *CI*]	*EC*_10_ (µg/mL)	*EC*_90_ (µg/mL)	*R* ^2^
AOP1 (intracell ROS)	72.02 [57.95–112.15]	15.80 [8.631–23.56]	328.3 [183.4–1218]	0.9787
ARE-luciferase	ND (repression effect)	ND (repression effect)	ND (repression effect)	ND
CAA (mb ROS)	9545 [7552–12,820]	1982 [1254–2857]	45,970 [27,120–101,200]	0.9697
ORAC (cell-free)	2.755 [2.447–3.096]	0.798 [0.589–1.045]	9.513 [7.374–12.97]	0.9866

## Data Availability

The data presented in this study are available on request from the corresponding author.

## Supplementary Materials

The following supporting information can be downloaded at: https://www.mdpi.com/article/10.3390/ijms262210827/s1.

## Abbreviations

The following abbreviations are used in this manuscript:

ARE	Antioxidant Response Element
AOP1	Anti Oxidant Power 1
CAA	Cellular Antioxidant Assay
Keap1	Kelch-like ECH-associated protein 1
Nrf2	Nuclear factor erythroid 2-related factor 2
RNS	Reactive Nitrogen Species
ROS	Reactive Oxygen Species
