# Supplementary material for: Tart Cherry (Prunus cerasus) Extract Exerts High Intracellular ROS Scavenging Activity and Repression of ARE (Antioxidant Response Element) Pathway in Human Hepatocytes
Source: Int J Mol Sci. 2025 Nov 7;26(22):10827. doi: 10.3390/ijms262210827 (PMC12652356; doi:10.3390/ijms262210827)
Supplement: Supplementary file 1 [file ijms-26-10827-s001.zip › ijms-3949446-supplementary.pdf]

Table S1 - Phenolic composition of *P. cerasus* (CherryCraft®)

P. cerasus (CherryCraft®)

Total polyphenols	Folin	196.90	g/kg
Anthocyanins	Spectro	32.94	g/kg
Phenolic acids	HPLC		
Chlorogenic acid		1.30	g/kg
Neochlorogenic acid		0.93	g/kg
Flavonols	HPLC		
Rutin		2.71	g/kg
Isoquercitrin		0.18	g/kg
Kampferol-3-rut		0.44	g/kg
Isorhamnetin-3-rut		1.04	g/kg
Proanthocyanidins procyanidin B2		3.96	g/kg

HPLC analysis of *P. cerasus* (CherryCraft®)

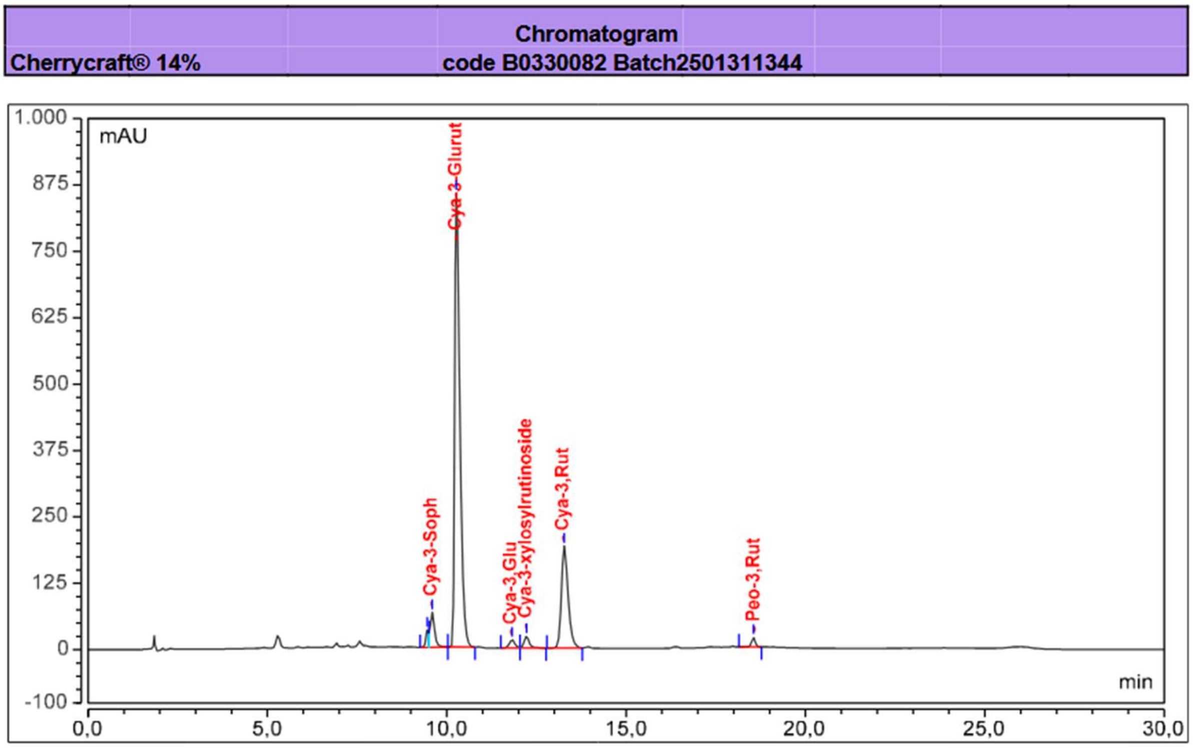

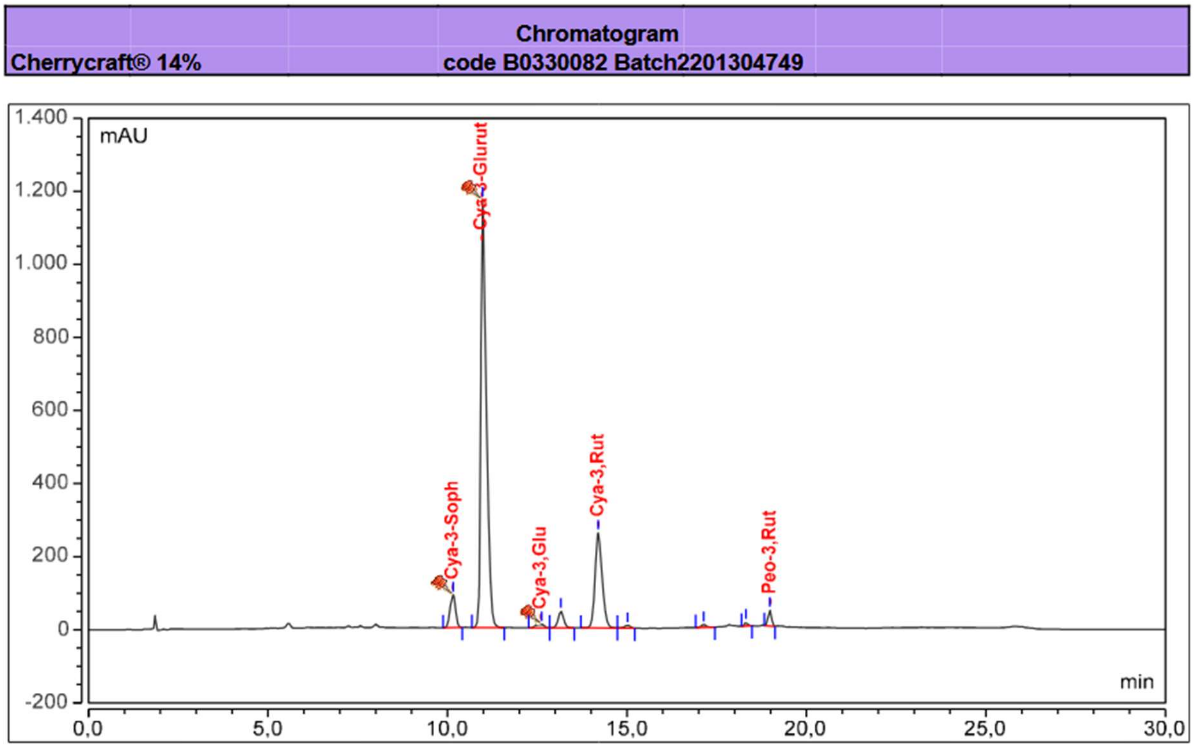

Figure S1. *HPLC chromatogram of CherryCraft® 14% tart cherry (Prunus cerasus) extract on two different batches.* The chromatogram displays the anthocyanin profile of the standardized extract, with major peaks identified as cyanidin-3-glucosylrutinoside (Cya-3-Glurut), cyanidin-3-sophoroside (Cya-3-Soph), cyanidin-3-glucoside (Cya-3-Glu), cyanidin-3-rutinoside (Cya-3-Rut), and peonidin-3-rutinoside (Peo-3-Rut). Detection was performed at 520 nm over a 0–30 min retention time.
